# Ectopic Expression of *PtoMYB74* in Poplar and Arabidopsis Promotes Secondary Cell Wall Formation

**DOI:** 10.3389/fpls.2018.01262

**Published:** 2018-10-09

**Authors:** Chaofeng Li, Xiaodong Ma, Hong Yu, Yongyao Fu, Keming Luo

**Affiliations:** ^1^Key Laboratory of Adaptation and Evolution of Plateau Biota, Northwest Institute of Plateau Biology, Chinese Academy of Sciences, Xining, China; ^2^University of Chinese Academy of Sciences, Beijing, China; ^3^School of Ecology and Environmental Resources, Qinghai University for Nationalities, Xining, China; ^4^School of Life Sciences, Southwest University, Chongqing, China

**Keywords:** *Populus*, R2R3-MYB, transcription factors, secondary cell wall, transgenic, genetic

## Abstract

In vascular plants, R2R3-MYB transcription factors are important regulators of secondary cell wall formation. Although 192 annotated R2R3 MYB genes were identified in the poplar genome, only a few members were characterized to participate in the regulation of the secondary cell wall biosynthesis. In this paper, we identify an R2R3-MYB transcription factor, *PtoMYB74*, which is predicted to be an ortholog of *Arabidopsis AtMYB61*, a transcription activator that regulates the secondary cell wall formation, lignin biosynthesis, stomatal aperture, and the mucilage of seed coat. *PtoMYB74* is mainly expressed in the stems, especially in the xylem tissues and organs. *PtoMYB74* as a transcriptional activator is localized to the nucleus. The overexpression of *PtoMYB74* increased the secondary cell wall thickness of vessels in transgenic poplar and changed the secondary cell wall compositions. The expression levels of lignin and cellulose biosynthetic genes were elevated in the transgenic poplar overexpressing *PtoMYB74* compared to the wild type, while there was no change in the xylan biosynthetic genes. Transcriptional activation assays demonstrated that *PtoMYB74* could activate the promoters of structural genes in the lignin and cellulose biosynthetic pathways. Taken together, our data show that *PtoMYB74* positively regulates the secondary cell wall biosynthesis in poplar.

## Introduction

Wood, which is mainly composed of a secondary cell wall, is utilized for a wide range of applications, such as wood pulping, paper making, house construction, biofuels, and bio-functional coating (Pilate et al., 2004). The secondary cell wall, which develops from beneath the primary cell wall, is mainly made of xylan, lignin, and cellulose (Douglas, 1996; Boudet, 2000). Owing to the massive economic values of wood, understanding the molecular mechanism of the secondary wall formation will be helpful for the improvement of wood quality through genetic engineering (Kubo et al., 2005).

In *Arabidopsis thaliana*, genetic and molecular analyses have identified a number of NAC and MYB transcription factors involved in the secondary wall formation, and recently an NAC-MYB-mediated transcriptional network regulating the secondary cell wall formation has been constructed (Zhong and Ye, 2014). In this complicated network, a couple of secondary-wall-related NAC transcription factors, including *SND1, NST1/2/3*, and *VND1/2/3/4/5/6/7* have been characterized as master switches that control many secondary wall-associated genes (Zhong et al., 2006; Mitsuda et al., 2007; Ohashi-Ito et al., 2010; Yamaguchi et al., 2011; Zhong and Ye, 2015). These *NAC* genes can directly or indirectly promote the expression of a great number of *MYB* genes (Zhong and Ye, 2015). Among these downstream target genes, *AtMYB58*/63/85 have been demonstrated as lignin-specific transcription factors (Zhou et al., 2009; Nakano et al., 2010), while *AtMYB26/32/41/4*4*/46/61/83/103* are shown to participate in the biosynthesis of secondary cell wall (Jin et al., 2000; Preston et al., 2004; Yang et al., 2007; Ko et al., 2009; McCarthy et al., 2009; Romano et al., 2012; Öhman et al., 2013; Kosma et al., 2014). In addition, Arabidopsis *MYB26* is also identified to up-regulate the expression of *NST1* and *NST2* genes (Yang et al., 2007; Ko et al., 2014).

In the past decade, it has been well-demonstrated that the regulation of the secondary wall biosynthesis by the NAC master switches is a conserved mechanism in vascular plants, and similar transcriptional regulatory networks have been established in woody plants, including *Eucalyptus gunnii*, spruce (*Picea glauca*), and poplar (Ohtani et al., 2011; Li et al., 2012). In poplar, six pairs of SND, VND, and NST homologs (PtrWNDs/PtrVNSs), such as *PtrWND2B*/*6B* and *PtrVNS12/16*, were identified to activate the expression of the secondary wall biosynthetic genes and induce ectopic deposition of lignin, xylan, and cellulose (Zhong et al., 2010, 2011; Ohtani et al., 2011; Li et al., 2012). These NAC proteins directly control the expression of several downstream genes, such as *PtrMYB2/3/20/21*, which are considered as the second-level master switches (Bomal et al., 2008). Dominant repressed expression of the MYB master switch genes led to a decrease in the secondary wall thickness in the woody tissues of transgenic plants (Patzlaff et al., 2003; Goicoechea et al., 2005; Bomal et al., 2008; McCarthy et al., 2010; Zhong et al., 2010, 2011), indicating their regulatory roles in secondary wall formation. In addition to the MYB genes mentioned above, a number of other MYB transcription factors have been shown to promote the up-regulation of the expression of some biosynthetic genes for xylan, lignin, and cellulose, thereby suggesting the complexity of the transcriptional regulatory network of wood formation (Zhong et al., 2011; Zhong and Ye, 2015). For example, the ortholog of *AtMYB58/63, PtrMYB28*, could activate the promoter activities of the lignin biosynthesis genes (Zhong and Ye, 2009). Overexpression of *PtrMYB152*, an ortholog of *AtMYB43*, improved lignin biosynthesis in transgenic Arabidopsis (Li et al., 2014). In our previous studies, *PtoMYB92* has been demonstrated to be a transcriptional activator of secondary wall biosynthetic pathway in *Populus tomentosa* (Li et al., 2015). The ectopic expression of *PtoMYB216* resulted in an increase of lignin content and changes in the xylan and cellulose components (Tian et al., 2013).

*AtMYB61*, an R2R3-MYB gene, is identified as a regulatory factor of plant growth and development (Romano et al., 2012). For example, *AtMYB61* controlled the stomatal aperture in response to light signals and diurnal cycle (Liang et al., 2005), and positively regulated the mucilage in its seed coat (Penfield et al., 2001). Recently, other studies also revealed that *AtMYB61* is involved in the regulation of xylem formation and lateral root development (Newman et al., 2004). Previous studies have shown that *PtMYB8*, the *Pinus taeda* ortholog of Arabidopsis *AtMYB61*, participates in the secondary wall formation as a positive regulator (Bomal et al., 2008). In this paper, we have characterized the function of *PtoMYB74*, an ortholog of *AtMYB61*, in the secondary cell wall biosynthesis in poplar. *PtoMYB74* is preferentially expressed in the stems, especially the xylem tissues. Overexpression of *PtoMYB74* in poplar and Arabidopsis resulted in a change of secondary cell wall thicknesses in vessels, and significantly increased the contents of lignin and cellulose. Furthermore, we demonstrated that *PtoMYB74* could activate the expression of some poplar key enzyme genes (*C3H3, C4H2, CCR2, CCOAOMT1, F5H2*, and *CesA2B*). These results suggest that *PtoMYB74* should play an important role in the positive regulation of secondary wall biosynthesis in poplar.

## Materials and Methods

### Plant Materials and Growth Conditions

The seeds of *Arabidopsis thaliana* (ecotype Columbia) were germinated and cultured on glass culture dishes containing MS medium (containing 30 g/L sucrose and 8.5 g/L Agar) for 2 weeks (Murashige and Skoog, 1962). The seedlings were transferred into nutrient soil for further growth in an illumination incubator with a culture condition of 20–23°C, 16/8 h light/dark cycle, 555 μmol m^-2^ s^-1^ supplemental light, and 80% humidity. The nutrient soil is a mixture of peat (Klasmann-Deilmann, Germany), vermiculite (Sanbao, China), and perlite (Longshan, China) in the proportion 6:3:1.

Poplar plants (*Populus tomentosa* Carr. clone 741) were transplanted from the culture dishes to customized pots and grown in the greenhouse. The conditions for seedlings culture in the greenhouse were 23–25°C, 16/8 h light/dark cycle, 278 μmol m^-2^ s^-1^ supplemental light, and 60% humidity (Li et al., 2015).

### Cloning of Full-Length *PtoMYB74* cDNA From *P. tomentosa*

Total RNA was extracted from the poplar leaves and cDNA was synthesized by reverse transcript polymerase chain reaction (RT-PCR). According to the sequence of *PtrMYB74* (XM_002321591.2/Potri.015G082700.1), the cDNA fragment (1116 bp) of *PtoMYB74* was amplified with gene-specific primers (**Supplementary Table S1**) and inserted into the cloning vector pMD19-T (TaKaRa) with the DNA Ligation Kit Solution I (TaKaRa). After sequencing, the fragment was subcloned into the plant binary vector pCXSN (Chen et al., 2009; **Supplementary Figure S1**). The resulting construct, pCXSN*-PtoMYB74*, was transferred into *Agrobacterium tumefaciens* stain EHA105 via the freeze-thaw method.

### Phylogenetic Analysis of *PtoMYB74*

For phylogenetic analysis, the amino acid sequences of R2R3 MYB genes, known to participate in the secondary wall formation, were acquired by the basic local alignment search tool (BLAST^1^). The multiple sequence alignment was then carried out with the DNAMAN8 (Altschul et al., 1990) software. The phylogenetic tree was constructed with the MEGA 6.06 software using the neighbor-joining (NJ) method with 2500 bootstrap replications under the p-distance model.

### RNA Extraction and RT-qPCR Analysis

Total RNA (TRIzol-extracted) was separately extracted from the Arabidopsis stems and various poplar tissues (including the young leaf, old leaf, root, stem, phloem, xylem, and petiole), and then genomic DNA was removed from the extracted RNA by DNAse treatment. The bark was separated from the poplar stem after girdling, and the phloem was obtained after scraping off all the epidermis and periderm. Meanwhile, the xylem was obtained after cutting away the pith from the rest of the stem. The first strand of cDNA was then synthesized by RT-PCR according to the manufacturer’s instructions (PrimeScript^TM^ RT Master Mix, TaKaRa). The RT-qPCR assay was performed as described previously (Nolan et al., 2006), and the 2^-ΔΔCt^ method was used to analyze the relative changes in gene expression (Livak and Schmittgen, 2001). RT-qPCR was performed using the Novostar-SYBR Supermix (Novoprotein, Shanghai, China) in a 96-well plate with the Thermal Cycler Dice Real Time System TP800 (TaKaRa, Japan) machine, and the Thermal Cycler Dice Real Time System Software Ver.5.00 was used for data collection and analysis. In each reaction (25 μl PCR reaction system), 12.5 μl Novostar-SYBR Supermix, 0.5 μM per primer pair, 15 ng of cDNA, and some amount of ultra-pure water were used. The initial denaturing conditions was 95°C for 30 s, followed by 40 cycles at 95°C for 5 s, and 60°C for 30 s. A melting curve was run after the PCR cycles, and the time was 95°C for 15 s, 60°C for 30 s, and 95°C for 15 s. The poplar *18S* rRNA was used as a control to calculate the relative expression level in WT and transgenic poplar (**Figures 2A, 5A, 7** and **Supplementary Figure S4**), while the Arabidopsis *UBC* gene was used as a reference gene in WT and transgenic Arabidopsis (**Figure 4B** and **Supplementary Figure S3**). The expression level of *Pto18S*/*AtUBC* was determined and set to 1.0. Three biological replicates were used for each sample and each RT-PCR reaction had three technical replicates. The tool Primer-BLAST^2^ was used to test the specificity of each primer pair, and the melting curve of each primer pair showed one single peak and a specific single product. The standard curve was used to test the amplification efficiency of each primer pair, and each primer pair had an appropriate PCR amplification efficiency (90.7–97.3%). The description of the RT-qPCR data followed all the MIQE-guidelines (Bustin et al., 2009).

### Semi-Quantitative RT-PCR Assays

The reverse transcribed cDNA samples (from Trizol-extracted Arabidopsis RNA) were used for semi-quantitative RT-PCR, and the *AtUBC* gene was used as an internal control. The conditions for semi-quantitative RT-PCR included an initial denaturation step at 94°C for 5 min, 28 cycles of 94°C for 45 s, 56°C for 45 s, and 72°C for 90 s, and an extension step at 72°C for 10 min. The amplification products were resolved by 1% (w/v) agarose gel electrophoresis and visualized with EB (ethidium bromide) under UV light.

### Subcellular Localization of *PtoMYB74*

The coding sequence (CDS) of *PtoMYB74* was amplified with gene-specific primers (**Supplementary Table S1**) from the full-length cDNA, and ligated to the binary vector pCX-DG (Chen et al., 2009) which contains a *GFP* reporter gene. The resulting construct containing the GFP and the full-length *PtoMYB74* CDS under the control of *CaMV 35S* promoter was infiltrated into the epidermal cells of tobacco leaves as described previously (Sparkes et al., 2006) and cultured in dark for 24 h. After 3 days, the GFP signal in the infected tobacco leaves was detected using an Olympus FV2000 confocal microscope at 488 nm laser.

### Yeast Activation Assay

To examine the *PtoMYB74* transcription activity, the *PtoMYB74* CDS was cloned into the vector pGBKT7. The resulting construct pGBKT7-PtoMYB74 was transformed into the yeast strain *Saccharomyces cerevisiae* Gold2 through the PEG-LiAc method (Zaragoza et al., 2004), while the pGBKT7 empty vector was used as a negative control. The transformants were grown on a synthetic dropout (SD) medium lacking tryptophan (Trp). The positive clones were then transferred into the SD medium lacking Trp, histidine (His), and adenine (Ade) for transcriptional activation activity analysis by staining with X-α-gal.

### Overexpression of *PtoMYB74* in Arabidopsis and *P. tomentosa*

The pCXSN-*PtoMYB74* construct was transformed into 30-day-old Arabidopsis plants by the floral dip method (Zhang et al., 2006). Transgenic plants were selected on ½ MS medium containing 50 mg/L hygromycin (Hyg) and 400 mg/L cefotaxime (Cef).

For poplar transformation, the pCXSN-*PtoMYB74* vector was transformed into *P. tomentosa* by the leaf disk method, as described previously (Jia et al., 2010). The transgenic plants were cultured on the woody plant medium (WPM; McCown and Lloyd, 1981) with 9 mg/L Hyg, 400 mg/L Cef, 30 g/L sucrose, and 8.5 g/L Agar. The survived seedlings were characterized by PCR and RT-qPCR. The positive transgenic plants were transferred into the soil and grown in the greenhouse.

### Isolation and Analysis of *PtoMYB74* Promoter

According to the genomic sequence of *PtrMYB74* (Potri. 015G082700.1^3^), the upstream flanking sequence (1500 bp) was used to design the gene-specific primers for *PtoMYB74* Promoter. The promoter fragment (1562 bp) of *PtoMYB74* was isolated from the *P. tomentosa* genomic DNA with the gene-specific primers listed in **Supplementary Table S1**. The first single-base of 5′UTR sequence (307 bp) was set as the transcriptional start site. The fragment was ligated to the binary vector pCX-GUS-P (Chen et al., 2009) containing a *GUS* reporter gene. The resulting vector was transformed into *A. tumefaciens* EHA105 strain for poplar transformation. The transgenic seedlings were used for GUS staining performed as instructed by Jefferson (1987).

### Transcriptional Activation Assay

The pCXSN-*PtoMYB74* vector was used as an effector construct. The promoter fragments of the *CCOAOMT1, CCR2, C3H3, C4H2, F5H2, GT8D, GT43B*, and *CesA2B* genes were amplified by the gene-specific primers listed in **Supplementary Table S1**, respectively, and then inserted into the reporter vectors to drive the *GUS* reporter gene. The co-transfection of reporter and effector constructs was performed in the leaves of *Nicotiana benthamiana* for transient expression by the agroinfiltration method (Sparkes et al., 2006). The GUS activity was measured using the 4-MUG (4-methylumbelliferyl-β-D-glucuronide) assay (Chen et al., 2005). After 3 days of infiltration, the tobacco leaves (100 mg each sample) were dealt with the freeze grinding technology and extracted with 1 ml GUS extraction buffer (50 mM sodium phosphate buffer, pH 7.0, 10 mM EDTA, 0.1% SDS, 0.1% β-mercaptoethanol, 0.1% triton-X 100) and then centrifuged (12000 rpm, 5 min, 4°C). After that, 100 μl of the supernatant was mixed with 900 μl MUG assay buffer (GUS extraction buffer containing 10 mM MUG) and incubated at 37°C for 60 min. The reaction was terminated by mixing 200 μl of aliquots with 800 μl 200 mM Na_2_CO_3_. The fluorescence intensity was measured and recorded in a Hitachi F-7000 fluorospectrophotometer with excitation at 365 nm and emission at 455 nm. The GUS activity was calculated as p mol MU mg min^-1^ protein^-1^.

### Scanning Electron Microscopy (SEM)

Transverse stem sections were hand-cut from different internodes of poplar plants with a razor blade. A Phenom Pure FEI SEM was used to examine the hand-cut sections, and the Revolution 1.6.1 software was employed to measure the radial width of xylem (μm) and the vessel cell wall thickness (μm).

### Histochemical Staining

Histochemical staining and observation of the stem cross-sections of Arabidopsis and poplar plants for lignin detection was performed as previously described (Chai et al., 2014; Li et al., 2015). In Phloroglucinol-HCl staining, after treatment with 37% HCl for 30 s, the sections were stained with 1.0% (w/v) phloroglucinol for 30 s (Li et al., 2015). In Toluidine Blue O (TBO) staining, the sections were stained with 1.0% (w/v) TBO for 30 s immediately (Chai et al., 2014). The stem cross-sections were then observed under a Zeiss Axio microscope (Zeiss, Oberkochen, Germany). Meanwhile, the stem cross-sections were dissected transversely with razor blades and directly observed under a scanning electron microscope (Phenom Pure FEI, United States) following the manual’s recommendations.

### Chemical Analysis of Secondary Cell Wall Components

The components of the secondary wall (lignin, cellulose, and xylan) were measured as previously described (Li et al., 2015). Briefly, the Klason and acetyl bromide (AcBr) methods were used to measure the lignin content (Iiyama and Wallis, 1988; Dence, 1992), while the Van Soest method was used to determine the xylan and cellulose contents (Van Soest and Wine, 1967).

### Statistical Analysis

The statistical analysis of all the experimental data is performed with Student’s *t*-test program^4^. Significant differences among data were evaluated via one-way ANOVA. ^∗^*p* < 0.05; ^∗∗^*p* < 0.01.

### GenBank Accession Numbers

The accession numbers of genes in the paper are listed in **Supplementary Table S2**.

## Results

### Isolation and Characterization of *PtoMYB74* From *P. tomentosa*

**FIGURE 1:**
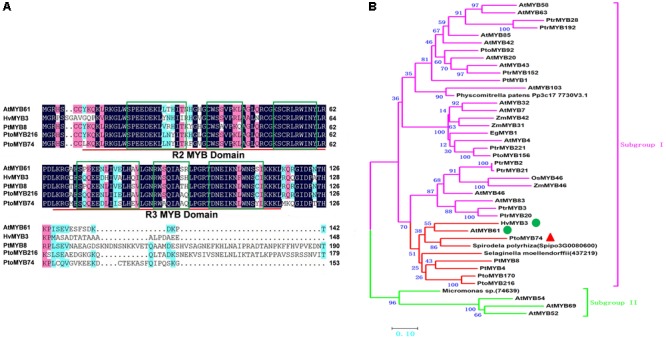
PtoMYB74 is highly conserved with many R2R3-MYB transcription factors. **(A)** Amino acid sequence alignment of the N-terminal region of PtoMYB74 with AtMYB61 (*A. thaliana*), HvMYB3 (*H. vulgare*), PtMYB8 (*P. taeda*), and PtoMYB216 (*P. tomentosa*). Sequences were aligned with the software of DNAMAN 8. Numbers on the left side refer to its sequence positions. The R2 and R3 repeats are indicated with red bars, and the Helix-Turn-Helix motifs are marked with green boxes. Blue and pink shades are used to show the identification and similarities of the amino acid residues, respectively. **(B)** Phylogenetic analysis of PtoMYB74 with selected R2R3-MYB transcription factors which are involved in secondary cell wall formation. Full-length of amino acid sequences were aligned with Clustal W, and the phylogenetic tree was constructed using MEGA 6.0 using the neighbor-joining method (2500 bootstrap replicates) and p-distance model. Bar = 0.1 substitutions per site. The Subgroup I is indicated with fuchsine bars, and the Subgroup II is marked with mint green bars. In the Subgroup I, *PtoMYB74* and its homologous genes are prominently displayed in the red bars. GenBank accession numbers of MYB transcription factors sequences are given in the **Supplementary Table S2**.

To investigate the function of poplar *MYB74* in poplar, the 1116 bp open reading frame sequence of *PtoMYB74*, encoding a protein with 371 amino acid residues, was isolated from *P. tomentosa* by RT-PCR. The predicted molecular weight of *PtoMYB74* was approximately 41.91 kDa, and its calculated isoelectric point (pI) was 6.44. The *PtoMYB74* protein consists of two conserved MYB domains at the N-terminal region, and each domain contains a Helix-Turn-Helix (HTH) motif (**Figure 1A**). The sequence analysis of *PtoMYB74* with other R2R3 MYB proteins showed that *PtoMYB74* and *PtoMYB216* (Tian et al., 2013) are a paralogous pair of genes with 89.74% identities in their R2R3 domain regions (**Figure 1A**). *PtoMYB74* also shares high amino acid identities with other R2R3 MYB transcription factors, such as *AtMYB61* (83.76%) in *A. thaliana, PtMYB8* (82.05%) in *P. taeda*, and *HvMYB3* (75.63%) in *Hordeum vulgare* L. (**Figure 1A**). The phylogenetic analysis revealed that *PtoMYB74* had close relationships with *PtMYB4, PtMYB8, HvMYB3, PtoMYB170, PtoMYB216*, and *AtMYB61* (**Figure 1B**), which are known to play important roles in the secondary cell wall formation (Wissenbach et al., 1993; Patzlaff et al., 2003; Bomal et al., 2008; Romano et al., 2012; Tian et al., 2013; Xu et al., 2017). Interestingly, we also found that *PtoMYB74* had close relationships with some lower plant species (*Selaginella moellendorffii* and *Spirodela polyrhiza*) (**Figure 1B**). The results suggest that the R2R3 MYB transcription factors are very conservative in the protein evolution of Plants.

### Expression Patterns of *PtoMYB74*

**FIGURE 2:**
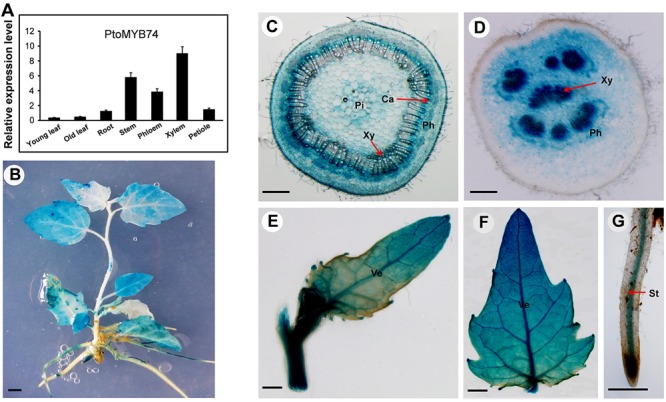
Express patterns of *PtoMYB74* in vascular blade, tissues, and organs of poplar. **(A)** RT-qPCR analysis of the relative expression levels of *PtoMYB74* in seven different tissues of wild-type poplar. The poplar *18S* rRNA was used as an internal reference. Error bars represent ± SD from three biological repeats. **(B–F)** The *PtoMYB74* gene promoter-driven GUS expression vector was introduced into *P. tomentosa* Carr. The GUS expression was detected in seedlings **(B)** and various tissues of *PtoMYB74:GUS* plants, including the stem **(C)**, petiole **(D)**, young leaf **(E)**, old leaf **(F)**, and root **(G)**. Ca, cambium; Ph, phloem; Pi, pith; St, stele; Ve, vein; Xy, xylem. The red arrow indicates the different tissues of poplar. Bars: **B** = 5 mm; **C,D** = 500 mm; **E,F** = 2 mm; **G** = 100 mm.

To determine the *PtoMYB74* expression pattern in poplar, the total RNA was extracted from different poplar tissues. The RT-qPCR analysis showed that the transcripts of *PtoMYB74* could be detected in all the organs and tissues, with higher expression levels in the stems, especially in the xylem tissues (**Figure 2A**).

To further investigate its spatial and temporal expression profiles, the 5′-flanking sequence of *PtoMYB74* was isolated and fused with a *GUS* reporter gene. After transformation, the histochemical staining results revealed that the GUS activity was detected in all organs and tissues of the transgenic pro*PtoMYB74:GUS* poplar plants (**Figure 2B**). *PtoMYB74* was predominantly expressed in vessels of different tissues (**Figures 2C–G**). In the stem, the cambium and xylem had strong GUS activities, while the pith and phloem exhibited relatively weak staining (**Figure 2C**). In the petiole, the *GUS* expression was observed in both xylem and phloem, but the GUS activity was relatively higher in the xylem (**Figure 2D**). In the young leaf, the *GUS* gene was predominantly expressed in the veins (**Figure 2E**), while the mature leaf had stronger GUS staining (**Figure 2F**). In the root, the GUS activity was mainly detected in the vascular cylinder (**Figure 2G**). Similar results were also obtained in transgenic pro*PtoMYB74:GUS* Arabidopsis (**Supplementary Figure S2**). These results are consistent with the RT-qPCR data above (**Figure 2A**), suggesting that *PtoMYB74* may participate in the secondary cell wall biosynthesis of poplar.

### Subcellular Localization of *PtoMYB74*

**FIGURE 3:**
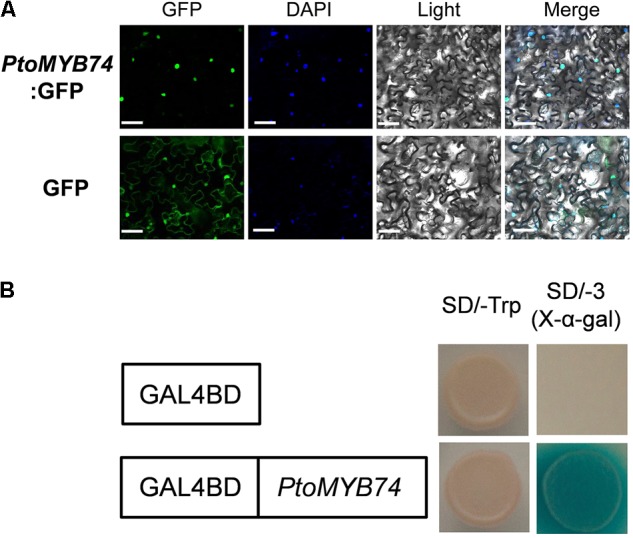
Subcellular localization and transcriptional activity analysis of PtoMYB74. **(A)** Confocal images of localization of GFP-PtoMYB74 in tobacco leaf epidermal cells. Bright field and fluorescent micrographs show the nuclear localization of GFP-PtoMYB74 and free GFP expressed both in nuclear and cytomembrane localization in tobacco (*Nicotiana benthamiana*) leaf epidermal cells. DAPI (4′,6-Diamidino-2-phenylindole dihydrochloride), a nuclear staining dye. Scale bars = 10 μm. **(B)** The transcriptional activity assays in yeast cells. The yeast carrying *GAL4BD-PtoMYB74* grew on a medium without Trp, His, and Ade (SD/–AHT) and induced the expression activity of X-α-Gal, while the negative control was not.

To examine its subcellular localization, the CDS of *PtoMYB74* was fused to the downstream of a *GFP* reporter gene driven by the *CaMV 35S* promoter. The resulting construct was introduced into the tobacco leaves by a transient expression system (Sparkes et al., 2006). As shown in **Figure 3A**, the GFP fluorescence was detected exclusively in the nuclei, which was co-localized by DAPI staining, thus indicating that *PtoMYB74* is a nucleus-localized protein.

### *PtoMYB74* Is a Transcriptional Activator

To investigate the transcriptional activity of *PtoMYB74*, we fused it with the GAL4 DNA binding domain (GAL4BD). The yeast recombinant transformants expressing the *GAL4BD:PtoMYB74* fusion protein grew well on the SD medium lacking Trp, His, and Ade, and was able to metabolize X-α-Gal to produce blue colonies (**Figure 3B**), indicating that *PtoMYB74* acts as a transcriptional activator in poplar.

### Overexpression of *PtoMYB74* Promotes Secondary Cell Wall Formation in Stems of Transgenic Plants

**FIGURE 4:**
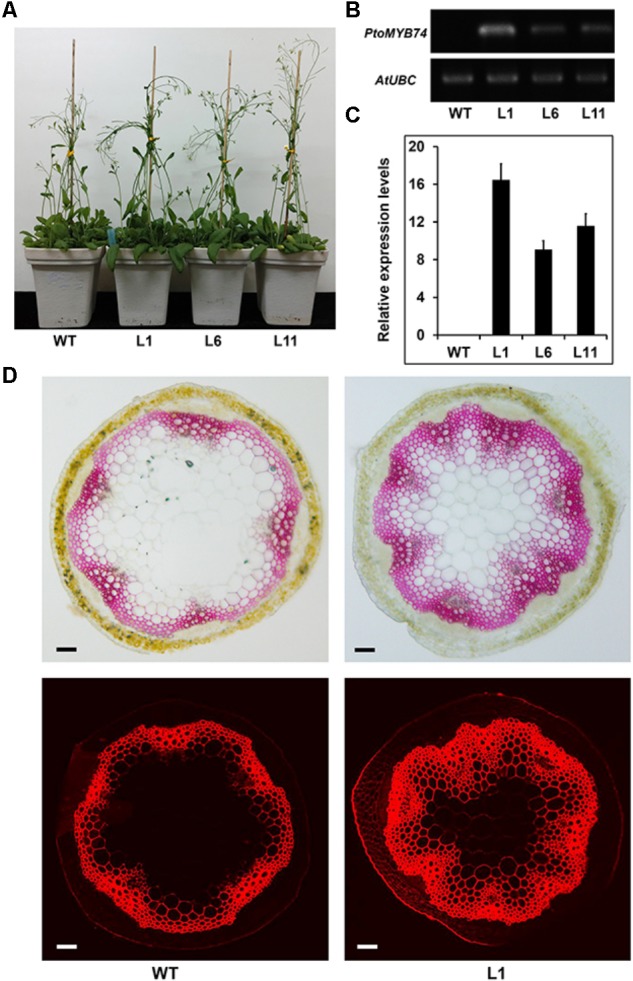
Phenotypes of transgenic Arabidopsis overexpressing *PtoMYB74*. **(A)** Representative 30-day-old wild-type and transgenic lines (L1, L6, and L11). **(B,C)** The relative expression levels of *PtoMYB74* in different lines (L1, L6, and L11). Semi-quantitative RT-PCR analyses of *PtoMYB74* in transgenic Arabidopsis plants **(B)**; Quantitative RT-PCR analyses of *PtoMYB74* in transgenic Arabidopsis plants **(C)**. **(D)** The phloroglucinol-HCl staining and lignin autofluorescence in wild-type and *PtoMYB74* overexpression line (L1). Bars: **C** = 500 μm.

To determine the role of *PtoMYB74* in the secondary cell wall biosynthesis, *PtoMYB74* was expressed in Arabidopsis under the control of the *CaMV 35S* promoter. More than 30 *PtoMYB74*-overexpression transgenic lines were generated and 3 homozygous lines (L1, -6, and -11) harboring a single copy of the transgene was selected for further experiments (**Figure 4A** and **Supplementary Figure S3**). The relative expression levels of *PtoMYB74* in transgenic plants were examined by semi-quantitative RT-PCR (**Figure 4B**) and RT-qPCR (**Figure 4C**). The *PtoMYB74* expression level was the highest in Line 1 (**Figures 4B,C**), and the Line 6 and Line 11 also had higher expression levels compared to the wild type Arabidopsis (**Figures 4B,C**). There was no significant difference in the phenotypes among the wild-type and *PtoMYB74*-overexpression transgenic plants (**Figure 4A**). Anatomical cross-sections of 40-day-old Arabidopsis plants were used for histochemical staining. The results showed that the overexpression of *PtoMYB74* caused stronger lignification in the xylem of transgenic plants, compared to the wild-type plants (**Figure 4D**). In *PtoMYB7*4-overexpression transgenic Arabidopsis plants, the lignin were also ectopically deposited in the walls of pith parenchyma cells (**Figure 4D**), which normally is not secondary walled and not lignified. Similar results were also obtained from lignin autofluorescence under UV light (**Figure 4D**). To count the xylem variations in the stems of *PtoMYB7*4-overexpression transgenic Arabidopsis plants, we measured the xylem radial width and the vessel cell wall thickness (**Supplementary Table S3**). The xylem width of *PtoMYB7*4-overexpression transgenic Arabidopsis plants had significantly increased by 50.83–108.33% compared to the wild-type plants, while the vessel cell wall thickness of *PtoMYB7*4-overexpression transgenic Arabidopsis plants had significantly increased by 24.14–62.65% compared to the wild-type plants (**Supplementary Table S3**). The results indicate that *PtoMYB74* is involved in the regulation of secondary wall deposition in the xylem tissues of transgenic Arabidopsis.

**FIGURE 5:**
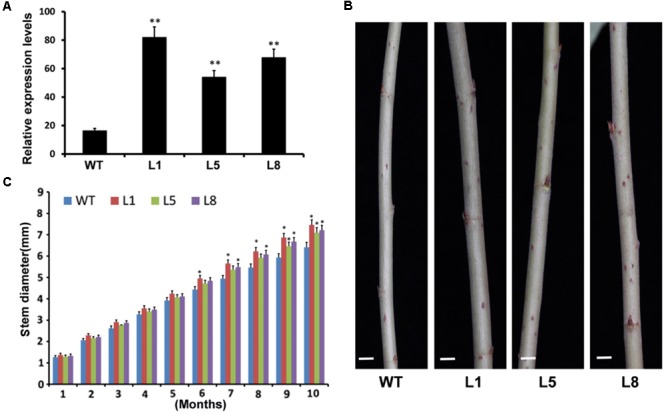
Phenotypes of transgenic poplar overexpressing *PtoMYB74*. **(A)** The expression levels of *PtoMYB74* in wild-type and three independent transgenic lines by RT-qPCR (after MIQE-guidelines). The poplar *18S* rRNA was used as an internal reference. **(B)** Stem microphotographs of the 10-month-old wild-type and *PtoMYB74* overexpression lines (L1, L5, and L8). **(C)** Stem diameters of wild-type and *PtoMYB74* overexpression lines (L1, L5, and L8). Error bars represent ± SD from three biological repeats. Student’s *t*-test: ^∗^*P* < 0.05; ^∗∗^*P* < 0.01. Bars: **C** = 5 mm.

**FIGURE 6:**
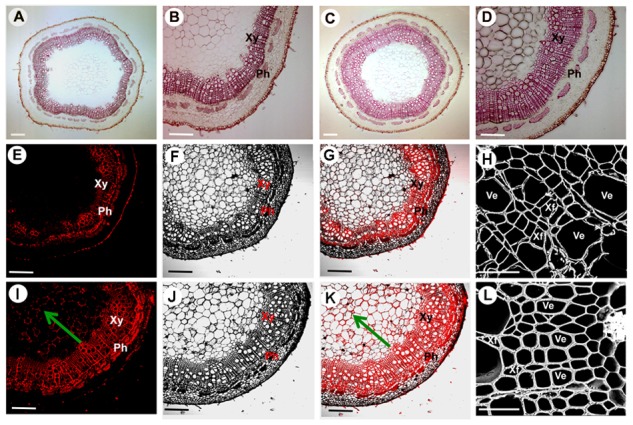
Effects of *PtoMYB74* overexpression on secondary cell wall thickening of poplar stems. **(A–D)** Stem sections from the sixth internode of 10-month-old wild-type and transgenic plants were stained with phloroglucinol-HCl. **(E–K)** Lignin auto-fluorescence of stem sections at the sixth internode in *PtoMYB74*-overexpressing transgenic and wild-type poplar under UV lights were detected by confocal fluorescence microscopy system. **(H,L)** Scanning electron micrographs of stem cross-sections at the sixth internode in *PtoMYB74*-overexpressing transgenic and wild-type poplars. Stem cross-sections used in these images were from the wild type **(A,B,E,F,G,H)** and transgenic poplars **(C,D,I,J,K,L)**. Ph, phloem; Xy, xylem; Ve, vessel; Xf, xylary fiber. The green arrow indicates the pith of stems. Scale bars: **A,C** = 500 μm; **B,D,E,F,G,I,J,K** = 200 μm; **H,L** = 10 μm.

The *35S:PtoMYB74* construct was further introduced into the Chinese white poplar (*P. tomentosa* Carr.). Over 25 putative transgenic plants were obtained and the integration of the transgene in the transgenic plants was confirmed by PCR with gene-specific primers of the hygromycin phosphotransferase (*HPT*) gene (**Supplementary Table S1** and **Supplementary Figure S4A**). Three independent transgenic lines (L1, -5, and -8) with high expression levels of *PtoMYB74* were used for further experiments (**Figure 5A** and **Supplementary Figure S4B**). The stem diameters of the transgenic plants overexpressing *PtoMYB74* displayed a significant increase compared to the wild-type plants (**Figures 5B,C** and **Supplementary Figure S5**). Phloroglucinol staining revealed a stronger lignification in xylem of transgenic *35S:PtoMYB74* lines (**Figures 6C,D**), compared to the wild type (**Figures 6A,B**). Meanwhile, more fiber cells in the sixth internode were observed in *PtoMYB74*-overexpression plants (17–23 layers) (**Figure 6D**), while only 9–13 layers of fiber cells were found in the wild-type plants (**Figure 6B**). Similar results were also obtained in other internodes of the transgenic and wild-type plants (**Supplementary Figure S6**). More lignin autofluorescence under UV light was detected in the transverse sections of transgenic *35S:PtoMYB74* lines (**Figures 6I–K**) as compared to that in the wild-type plants (**Figures 6E–G**). Interestingly, we also observed the ectopic deposition of lignin in pith cells of transgenic *35S:PtoMYB74* lines (**Figure 6I**). It is worth mentioning that we also observed the cambium in stems of wild type and *PtoMYB74*-overexpression poplar (**Supplementary Figure S7**). More cambium cell layers in the sixth internode was observed in *PtoMYB74*-overexpression plants (6–12 layers) (**Supplementary Figures S7A,C**), while only 3–7 layers of cambium cells were found in the wild-type plants (**Supplementary Figures S7B,D**). Furthermore, the xylem radial width and the vessel cell walls of transgenic lines overexpressing *PtoMYB74* were significantly thicker than that in the wild type (**Figures 6H,L** and **Table 1**). The xylem width of poplar transgenic *35S:PtoMYB74* lines had significantly increased by 12.90–33.47% compared to the wild-type plants, while the vessel cell wall thickness of poplar transgenic *35S:PtoMYB74* lines had significantly increased by 29.59–61.22% compared to the wild-type plants (**Table 1**). The phenotype analysis and statistical data in poplar are in line with that in Arabidopsis. These results showed that *PtoMYB74* positively regulated the secondary cell wall formation in poplar, especially the ectopic deposition of lignin.

**Table 1:** Radial width of xylem and cell wall thickness of vessels in stems of WT and *PtoMYB74* transgenic poplar plants.

Samples		Radial width of)	Vessel cell wall
		xylem (μm)	thickness (μm)
WT		3689.45 ± 104.21	0.98 ± 0.13
	L1	4926.46 ± 156.70^∗∗^	1.53 ± 0.19^∗∗^
*35S:PtoMYB74*	L5	4165.33 ± 117.66^∗^	1.27 ± 0.12^∗^
	L8	4571.81 ± 132.97^∗∗^	1.38 ± 0.11^∗^

*The results are given as means ± SD of three independent replicates. Asterisks indicate significant differences (Student’s t-test at ^∗^*P* < 0.05; ^∗∗^*P* < 0.01).*

To quantify the changes of secondary wall components, the lignin, cellulose, and xylan contents were determined in the stems of transgenic *35S:PtoMYB74* lines via both Klason and acetyl bromide (AcBr) methods. The results revealed that the Klason lignin contents in the stems of transgenic *35S:PtoMYB74* lines were significantly increased by 13.39–21.53% compared to the wild-type plants (**Table 2**). Similar results were also obtained from the AcBr method (**Table 2**). Moreover, overexpression of *PtoMYB74* appeared to have no significant effect on the xylan biosynthesis, while higher cellulose levels (10.13–12.46%) were detected in transgenic *35S:PtoMYB74* lines (**Table 2**).

**Table 2:** Secondary cell wall composition analysis of the stems in wild-type and *PtoMYB74* overexpression plants.

	WT	*35S:PtoMYB74*		
		L1	L5	L8
**Lignin**				
AcBr-soluble	22.56 ± 0.54	25.39 ± 0.74^∗^	23.82 ± 0.66^∗^	24.92 ± 0.62^∗^
Klason	19.97 ± 0.29	24.27 ± 0.58^∗^	22.59 ± 0.35^∗^	23.79 ± 0.46^∗^
Acid-soluble	2.78 ± 0.23	3.41 ± 0.49^∗^	2.88 ± 0.39	3.25 ± 0.49^∗^
Total lignin	22.75 ± 0.52	27.68 ± 1.07^∗^	25.47 ± 1.44^∗^	27.04 ± 0.95^∗^
**Polysaccharide**				
Xylan	15.48 ± 0.57	15.89 ± 0.46	14.96 ± 0.49	15.21 ± 0.63
Cellulose	42.36 ± 1.37	45.64 ± 1.97^∗^	44.15 ± 1.46^∗^	44.99 ± 1.88^∗^

*Plant materials were prepared from the stems of 10-month-old plants and determined by Klason, AcBr, and Van Soest methods. The results are given as means ± SD of three independent replicates. Asterisks indicate significant differences (Student’s t-test at ^∗^P < 0.05).*

### Overexpression of *PtoMYB74* Improves the Expression of Secondary Wall Biosynthetic Genes in Transgenic Poplar

**FIGURE 7:**
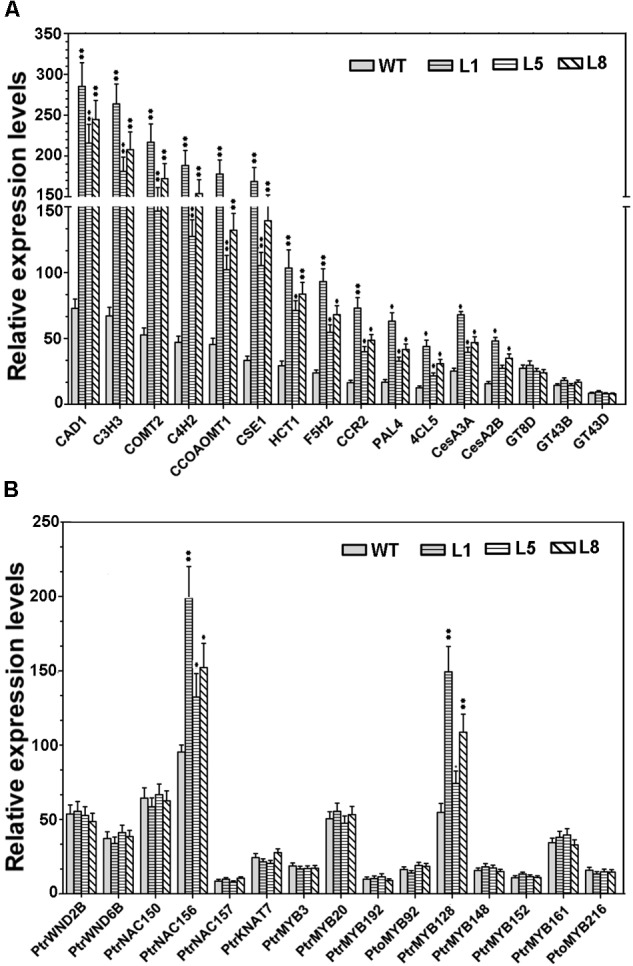
The expression of several secondary wall biosynthetic genes and secondary wall-associated transcription factors in the stems of wild-type and *PtoMYB74* overexpression poplar plants by RT-qPCR (after MIQE-guidelines). **(A)** Key enzyme genes in secondary cell wall biosynthetic pathway. **(B)** Transcription factors involved in the regulation of secondary cell wall biosynthesis. The poplar *18S* rRNA was used as an internal reference. Error bars represent ± SD from three biological repeats. Student’s *t*-test: ^∗^*P* < 0.05; ^∗∗^*P* < 0.01.

Given that the constitutive expression of *PtoMYB74* led to the ectopic deposition of lignin and secondary wall thickening in the stems of transgenic poplar, we further investigated whether the key enzyme genes involved in the secondary wall biosynthetic pathway were activated by *PtoMYB74.* The RT-qPCR analysis revealed that the relative expression levels of all the lignin synthetic genes (*CAD1, C3H3, COMT2, C4H2, CCOAOMT1, CSE1, HCT1, F5H2, CCR2, PAL1*, and *4CL5*) (Shi et al., 2010; Vanholme et al., 2013) and two cellulose synthetic genes (*CesA2B* and *CesA3A*) (Joshi et al., 2004; Djerbi et al., 2005) were elevated in the *PtoMYB74* overexpressing lines (**Figure 7A**), consistent with an increase of lignin and cellulose contents in the transgenic plants (**Table 2**). However, the expression of three xylan synthetic genes (*GT8D, GT43B*, and *GT43D*) (Lee et al., 2011) was not activated in the transgenic plants (**Figure 7A**). In addition, all of the secondary wall-associated NAC and MYB transcription factors, except *PtrNAC156* and *PtrMYB128*, were not induced in the transgenic lines compared to the wild-type plants (**Figure 7B**). These results suggested that *PtoMYB74* may positively regulate the secondary wall biosynthesis by activating the lignin and cellulose biosynthetic genes in poplar.

**FIGURE 8:**
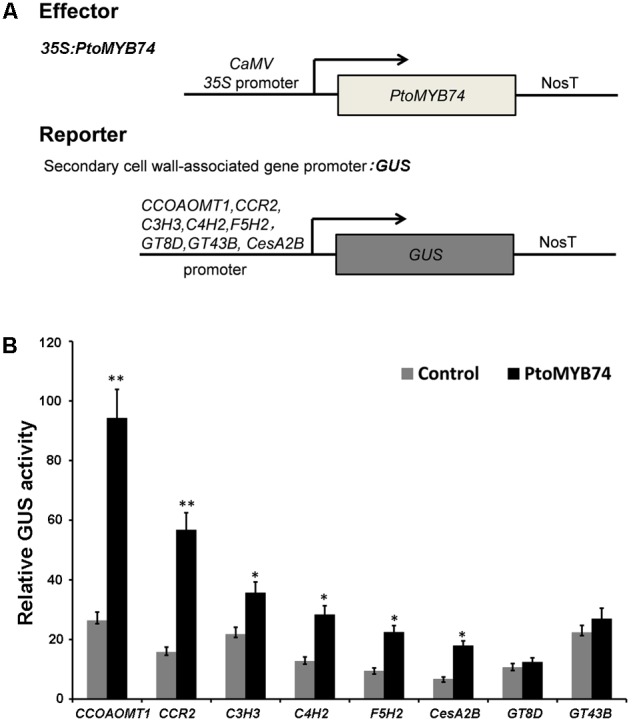
Activation of the promoters of some secondary cell wall biosynthetic genes by *PtoMYB74*. **(A)** Diagrams of the effector and reporter constructs used for transactivation analysis. The effector construct is the *PtoMYB74* cDNA driven by the *35S* promoter. The reporter constructs contain the GUS reporter genes driven by the promoters of some secondary cell wall biosynthetic genes, including *CCOAOMT1, CCR2, C3H3, C4H2, F5H2, GT8D, GT43B*, and *CesA2B*. **(B)** Transactivation analysis shows that *PtoMYB74* activated individually the *CCOAOMT1/CCR2/C3H3/C4H2/F5H2/CesA2B* promoter-driven expression of the GUS reporter gene. GUS activity in leaves transfected with the reporter gene alone was used as a control. Error bars represent ± SD of three biological replicates. Student’s *t*-test: ^∗^*P* < 0.05; ^∗∗^*P* < 0.01.

Transactivation assays were used to determine whether *PtoMYB74* is capable of activating the promoters of some key enzyme genes in the secondary wall biosynthetic pathway. Transient expression experiments in tobacco leaves showed that *PtoMYB74* could activate the promoters of biosynthetic genes for lignin (*CCOAOMT1, CCR2, C3H3, C4H2*, and *F5H2*) and cellulose (*CesA2B*) (**Figure 8**). In addition, the promoters of *GT8D* and *GT43B* were not activated by *PtoMYB74*. These results indicate that *PtoMYB74* functions as a transcription activator of secondary cell wall biosynthesis, and preferentially regulates lignin and cellulose biosynthesis during wood formation in poplar.

## Discussion

Wood formation is a very complex process that requires the coordinated and regulated expression of secondary cell wall biosynthesis in higher plants. A subset of the secondary wall biosynthetic genes, including *CAD, C3H, COMT, C4H, CCOAOMT, CSE, HCT, F5H, CCR, PAL, 4CL, CesA, CSLC*, and *GT*, have been isolated and characterized in various plant species (Djerbi et al., 2005; Betancur et al., 2010; Shi et al., 2010; Yin et al., 2010; Carroll et al., 2012; Rennie et al., 2012). The expression of these key enzyme genes was controlled by the secondary cell wall transcriptional regulatory network (Zhong et al., 2010; Zhong and Ye, 2014). In annual model plants, such as *A. thaliana* and tobacco, many NAC and MYB transcription factors have been identified to be involved in the regulation of the secondary cell wall formation through comprehensive molecular and genetic studies (Zhong and Ye, 2014). The transcriptional regulation mechanism of the secondary cell wall formation is more complicated and sophisticated in woody plants. For example, a number of R2R3-MYB transcription factors responsible for the secondary cell wall formation have been functionally characterized in poplar, such as *PtrMYB3, PtrMYB20, PtrMYB152, PtoMYB92, PtoMYB216*, and *PdMYB221* (McCarthy et al., 2010; Tian et al., 2013; Wang et al., 2014; Tang et al., 2015).

In the present study, we have isolated an R2R3 MYB transcription factor *PtoMYB74*, a homologous gene of Arabidopsis *MYB61*, and identified that it is involved in the transcriptional regulation of secondary cell wall biosynthesis. As shown in **Figure 1A**, *PtoMYB74* contains a highly conserved R2R3-MYB region, and shares high similarities with *AtMYB61* (Romano et al., 2012), *HvMYB3* (Wissenbach et al., 1993), *PtoMYB216* (Tian et al., 2013), and *PtMYB8* (Bomal et al., 2008). Phylogenetic analysis displayed that *PtoMYB74* also had a high homology with the MYB transcription factors of *Selaginella moellendorffii* and *Spirodela polyrhiza* (**Figure 1A**). The results indicated that the MYB transcription factors were very conservative in the cell wall formation of vascular plants. Expression pattern analysis showed that *PtoMYB74* is predominantly expressed in the vascular tissues (**Figure 2**), which are mainly made up of the xylem and phloem, thereby suggesting its potential roles in the transcriptional regulation of secondary cell wall biosynthesis. Overexpression of *PtoMYB74* not only significantly increased the cell wall thickness of the xylem vessels in the stems of Arabidopsis and poplar but also significantly increased the radial width of the xylem compared to that in the wild-type plants (**Figures 4–6, Table 1**, and **Supplementary Table S3**). Consistent with the morphological and anatomical observations, the contents of lignin and cellulose were elevated in the stems of the *PtoMYB74* overexpressing transgenic poplar, compared to the wild-type plants (**Table 2**). The lignin deposition in the parenchyma cells (**Figure 4D**) and piths (**Figures 4D, 6**) was also observed, and the phenotypes of the *PtoMYB74* overexpressing transgenic Arabidopsis were very similar to the mutants of *atelp-1* (Zhong et al., 2000) and *atwrky12-1* (Wang et al., 2010). *PtoMYB74* may also participate in the plants lignification process in response to wounding or infection (Zhong et al., 2000; Wang et al., 2010). However, the mechanism of poplar MYB transcription factors’ function in the parenchyma cells and pith cell wall formation is still unclear. Meanwhile, we also found that the xylem tissue of transgenic *35S:PtoMYB74* lines had more cell layers (**Figure 6**). Interestingly, the *PtoMYB74*-overexpression transgenic plants also had more layers in the cambium cells (**Supplementary Figure S7**). The results indicated that *PtoMYB74* can affect the cell division of cambial cells and differentiation into xylem vessels. Genome-wide expression profiling of xylem and phloem-cambium isolated from the root-hypocotyl of Arabidopsis showed that the class III HD-ZIP and KANADI transcription factors could function as regulators of radial patterning during secondary growth, and the G2-like, NAC, AP2, MADS, and MYB transcription factors may play important roles as regulators of xylem or phloem cell differentiation and activity (Zhao et al., 2005). However, further work about the roles of the NAC/MYB transcription factors in the xylem or phloem cell differentiation and activity has not been carried out. Similarly, the links between the NAC/MYB transcription factors (including *PtoMYB74*) and cambial cells differentiation into xylem vessels are still unclear. The research about cell division of cambial cells and differentiation into xylem vessels has been concentrated in the model of peptide signaling (Chen et al., 2014; Nieminen et al., 2015). It is worthy to take on the effects of the NAC/MYB transcription factors on the cell division of cambial cells and differentiation into xylem vessels during further research. Taken together, a subset of experimental results suggests that the poplar *PtoMYB74* may function as a positive genetic regulator to take part in the secondary cell wall biosynthesis during wood formation.

In previous studies, many MYB transcription factors have been shown to act as transcription activators of secondary cell wall biosynthesis (McCarthy et al., 2010; Tian et al., 2013; Zhong and Ye, 2014; Tang et al., 2015). The transcriptional activity assays showed that *PtoMYB74* encodes a nucleus-localized protein (**Figure 3A**) and activated the β-galactosidase reporter gene expression in the GAL4BD-based self-activation system (**Figure 3B**), indicating that it is localized to the nucleus and functions as a transcriptional activator. Overexpression of *PtoMYB74* in poplar increased the expression of secondary wall-associated key enzyme genes (*CAD1, C3H3, COMT2, C4H2, CCOAOMT1, CSE1, HCT1, F5H2, CCR2, PAL1, 4CL5 CesA2B*, and *CesA3A*) and two wood-formation-related transcription factors (*PtrNAC156* and *PtrMYB128*) (**Figure 7**). Moreover, the *CCOAOMT1, CCR2, C3H3, C4H2, F5H2*, and *CesA2B* promoters were activated by *PtoMYB74* in the tobacco transient expression system (**Figure 8**), indicating that these genes are possible direct targets of *PtoMYB74*. Further studies are required to test this speculation by chromatin immunoprecipitation assays or electrophoretic mobility shift assays. In addition, a previous study has reported that *AtMYB61* regulated the stomatal aperture and enhanced the drought tolerance in Arabidopsis (Romano et al., 2012). In our previous studies, the function of *PtoMYB216* has been heavily focused on manipulation of lignin biosynthesis (Tian et al., 2013), while the *PtoMYB170* positively regulates the lignin deposition during wood formation in poplar and confers drought tolerance in transgenic Arabidopsis (Xu et al., 2017). In this research, we have demonstrated that *PtoMYB74* not only promoted the lignin deposition in poplar and Arabidopsis but also increased the accumulation of cellulose. As the functional orthologs of *AtMYB61*, the three R2R3 MYB family members *(PtoMYB74, PtoMYB216*, and *PtoMYB170*) have functional redundancy in lignin biosynthesis and functional differentiation in stress response and substance metabolism. From the phylogenetic analysis and amino acid sequence alignment (**Figure 1**; Tian et al., 2013; Xu et al., 2017), we can learn that they are highly conserved in the N-terminal region, especially the R2R3-MYB domain. On comparing with the expression patterns of the three MYB transcription factors (**Figure 2**; Tian et al., 2013; Xu et al., 2017), we have found that they all have high expression levels in lignin-rich tissues (xylem and phloem). These should supply partial evidence for their functional redundancy in the lignin biosynthesis. The amino acid sequence alignment also shows that they are diverse and complicated in the C-terminal region (**Figure 1**; Tian et al., 2013; Xu et al., 2017), and these variations may sustain functional divergences. Promoter analysis showed that they also have various elements to respond to diverse stresses, except for AC element (**Figure 2** and **Supplementary Figure S2**; Tian et al., 2013; Zhong et al., 2013; Xu et al., 2017). Unlike *PtoMYB74* and *PtoMYB216, PtoMYB170* had higher expression levels in young leaves, and this may explain why *PtoMYB170* positively confers the drought tolerance connecting with promoter analysis (Xu et al., 2017). Apart from *PtoMYB216* and *PtoMYB170, PtoMYB74* also promotes the cellulose accumulation (**Table 2**). Promoter analysis showed that *PtoMYB74* has several elements to respond to cellulose synthesis, and the results of RT-qPCR and transcriptional activity analysis supply the data for this analysis (**Figures 7, 8**). *AtMYB61* also positively regulates the mucilage in its seed coat (Penfield et al., 2001), and a similar research is difficult for poplar because of its growth cycle.

In summary, this study has identified *PtoMYB74* as a poplar ortholog of *AtMYB61* that is involved in the secondary wall biosynthesis via the activation of key synthetic enzyme genes in the biosynthetic pathways of lignin and cellulose. Constitutive expression of *PtoMYB74* increased the secondary cell wall thickness of the xylem vessels in the stems of transgenic poplar and caused ectopic deposition of lignin and cellulose. Our results may provide some new information about the complex and sophisticated regulatory network of secondary wall biosynthesis in poplar, and help us better understand the wood formation.

## Author Contributions

KL and CL designed this work. CL and XM performed the experiments, analyzed the data, and drafted the manuscript. HY and YF analyzed the data and drafted the manuscript. KL revised the manuscript. All authors approved the final revision of the manuscript for publication.

## Conflict of Interest Statement

The authors declare that the research was conducted in the absence of any commercial or financial relationships that could be construed as a potential conflict of interest.

## Footnotes

1 https://blast.ncbi.nlm.nih.gov/Blast.cgi

2 https://www.ncbi.nlm.nih.gov/tools/primer-blast/

3 https://phytozome.jgi.doe.gov/pz/portal.html

4 http://www.graphpad.com/quickcalcs/ttest1.cfm
