# Supplementary material for: Ectopic Expression of PtoMYB74 in Poplar and Arabidopsis Promotes Secondary Cell Wall Formation
Source: Front Plant Sci. 2018 Oct 9;9:1262. doi: 10.3389/fpls.2018.01262 (PMC6191708; doi:10.3389/fpls.2018.01262)
Supplement: Supplementary file 1 [file Presentation_1.PDF]

Ectopic Expression of *PtoMYB74* in Poplar and Arabidopsis Promotes Secondary Cell Wall Formation

Authors: Chaofeng Li [a, b], Xiaodong Ma[a, b, c] and Keming Luo[a*]

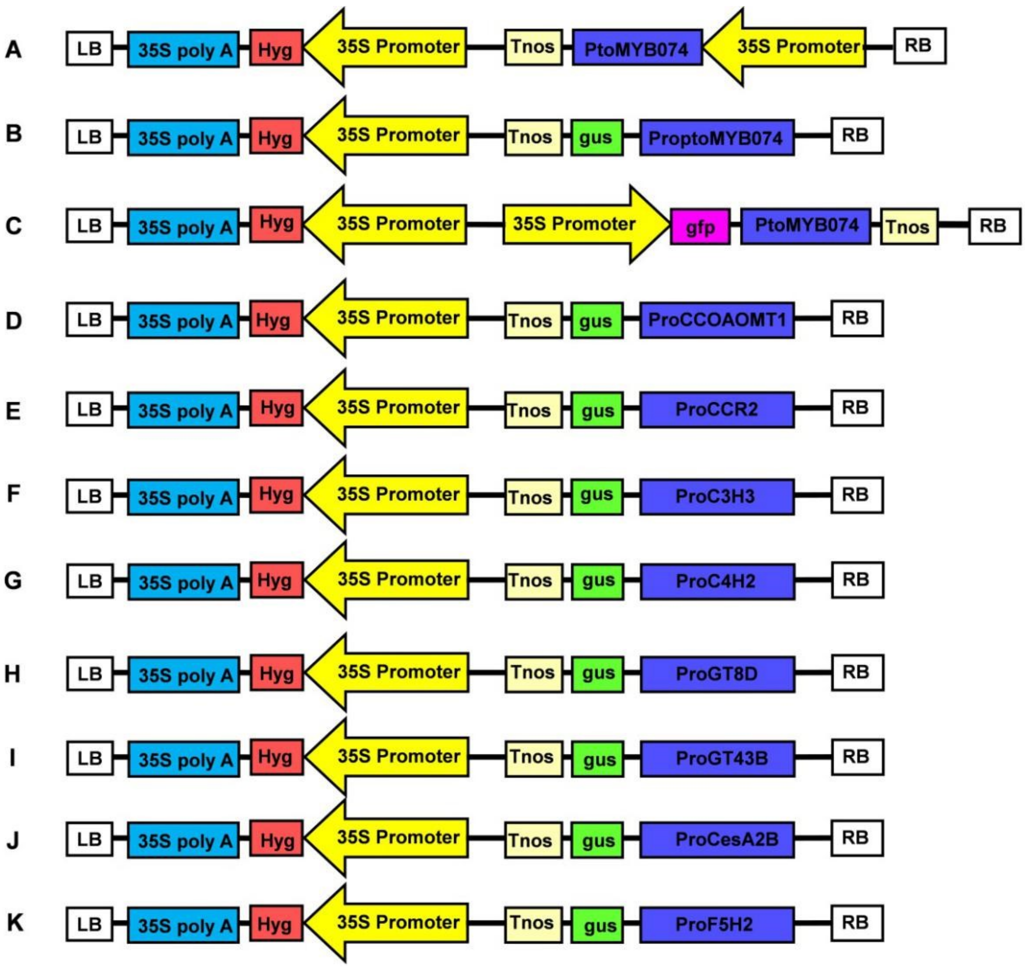

FIGURE S1| Schematic diagram of the vectors. LB: T-DNA left border; RB: T-DNA right border; 35S promoter: cauliflower mosaic virus promoter; 35S polyA: terminator of *CaMV 35S*; Tnos: terminator form nopaline synthase gene; Hyg: hygromycin phosphotransferase gene; GUS: β-glucuronidase; GFP: green fluorescent protein. (A) The binary vector for *PtoMYB74*-Overerpression in Arabidopsis and poplar. (B) The vector for promoter analysis. (C) The vector for subcellular localization. (D-K) Vectors for transactivation analysis.

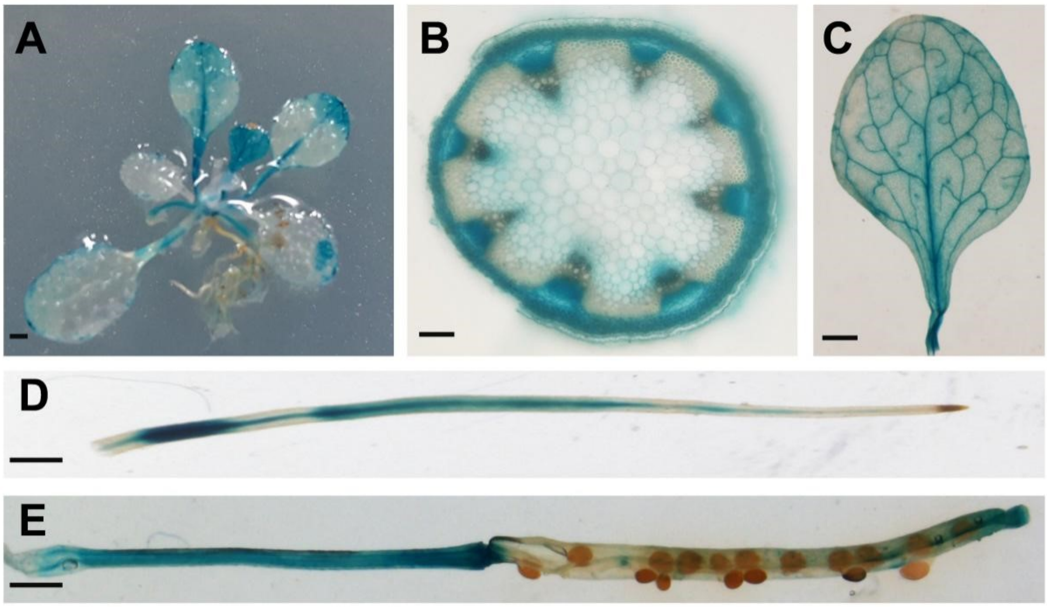

FIGURE S2| Express patterns of *PtoMYB74* in Arabidopsis. Seedling (A), Stem (B), Leaf (C), Root (D) and Silique (E). Bars: A =2 mm; B, C = 500 µm; D, E = 100 µm.

A

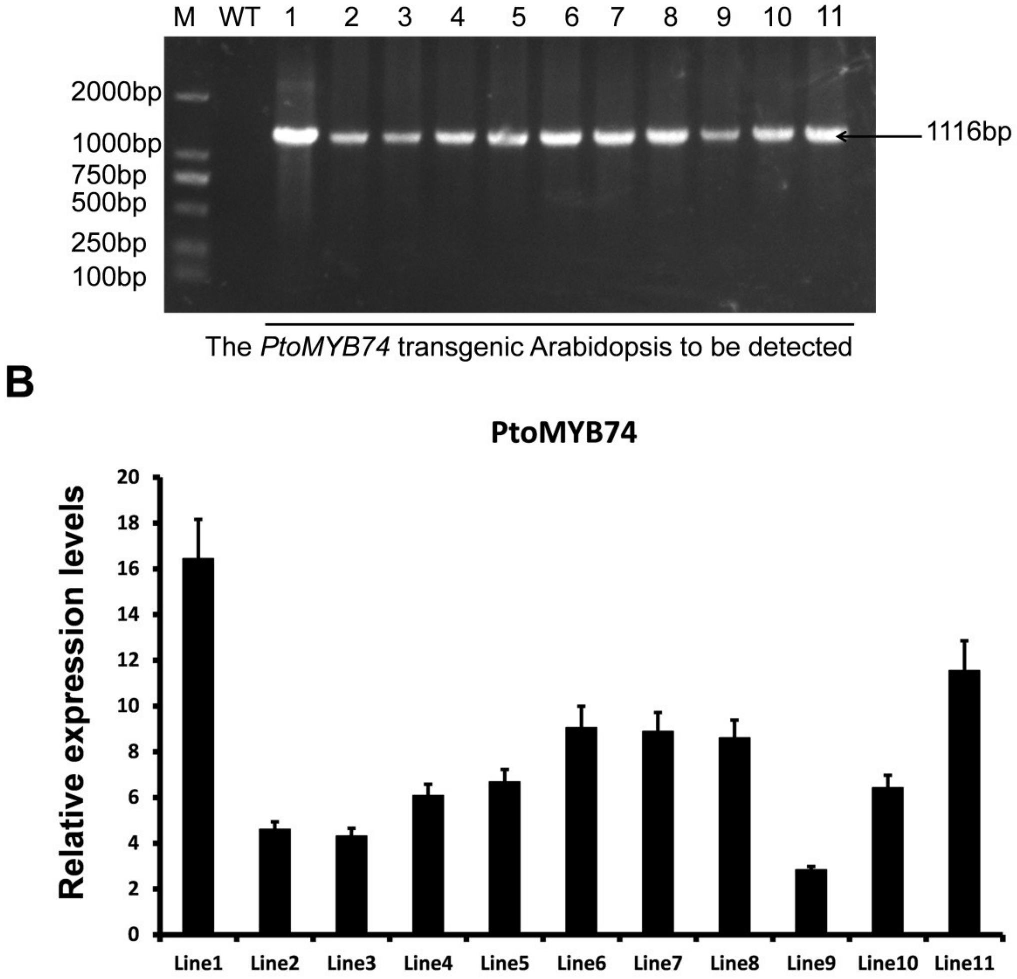

FIGURE S3| Identification of transgenic Arabidopsis plants. (A) PCR analysis of transgenic Arabidopsis plants. Genomic DNAs were isolated from hygromycin-resistant plants transformed with the *35S:PtoMYB74* vector. M, DL2000 DNA Marker; (B) The relative expression levels of *PtoMYB74* transcription factor in different lines. Quantitative RT-PCR analyses of *PtoMYB74* in transgenic Arabidopsis plants. Lines (1-11) refer to independent transgenic lines. The line 1, 6 and 11 had the higher expression levels, and were used for further analysis. Error bars represent ± SE from three biological repeats. Student's *t*-test:*, $P < 0.05$; **, $P < 0.01$.

A

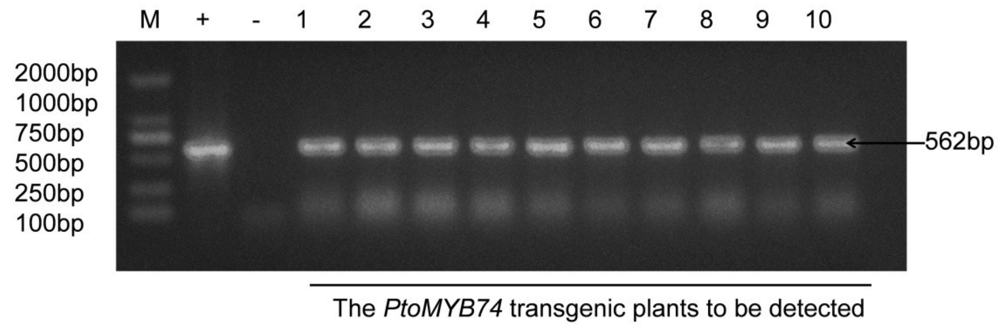

B

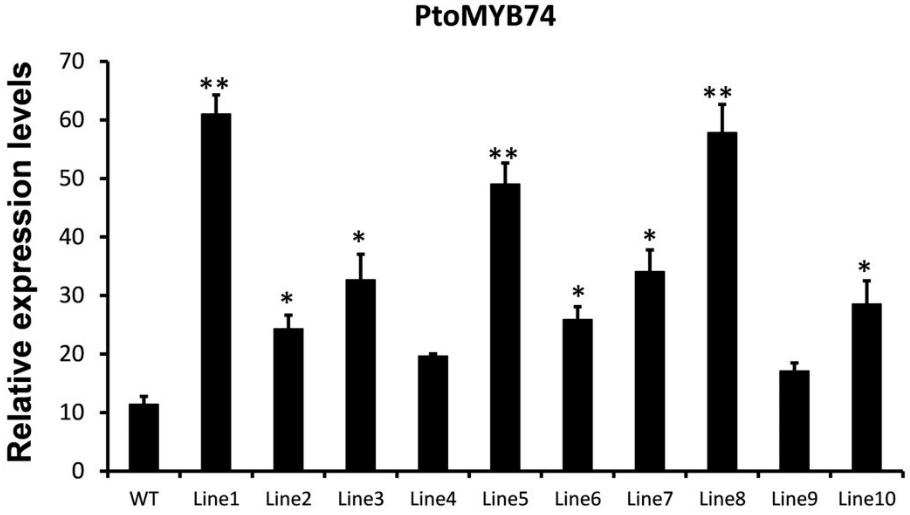

FIGURE S4| Identification of transgenic poplar plants. (A) PCR analysis of transgenic poplar plants. Genomic DNAs were isolated from hygromycin-resistant plants transformed with the *35S:PtoMYB74* vector. M, DL2000 DNA Marker; +, corresponding plasmid DNA (positive control); -, ddH_2O (negative control); (B) The relative expression levels of *PtoMYB74* in transgenic lines. Quantitative RT-PCR analysis of *PtoMYB74* in transgenic poplar plants. Lines (1-10) refer to independent transgenic lines. The line 1, 5 and 8 had the higher expression levels, and were selected for further analysis. Error bars represent ± SE from three biological repeats. Student's *t*-test:*, $P < 0.05$; **, $P < 0.01$.

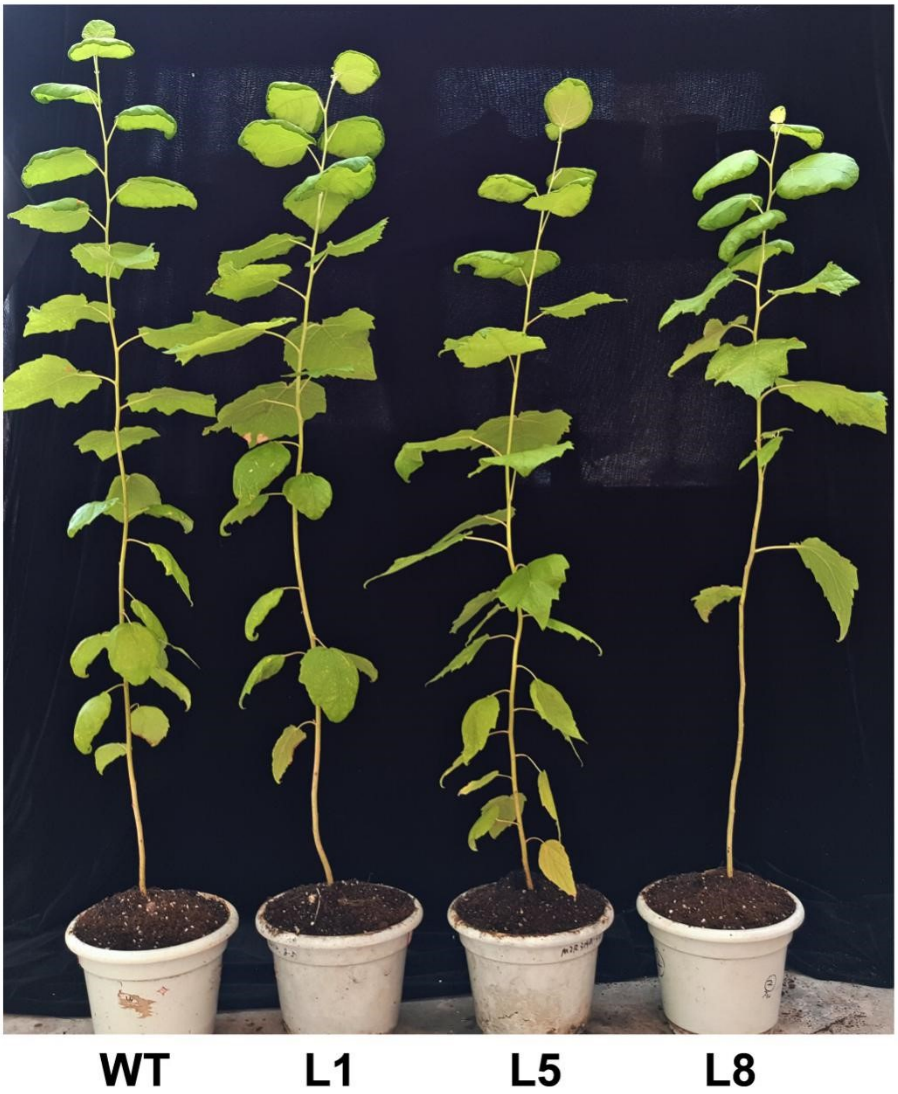

FIGURE S5| Phenotypes of transgenic poplar overexpressing *PtoMYB74*.. Lines (L1, L5 and L8) refer to independent transgenic lines.

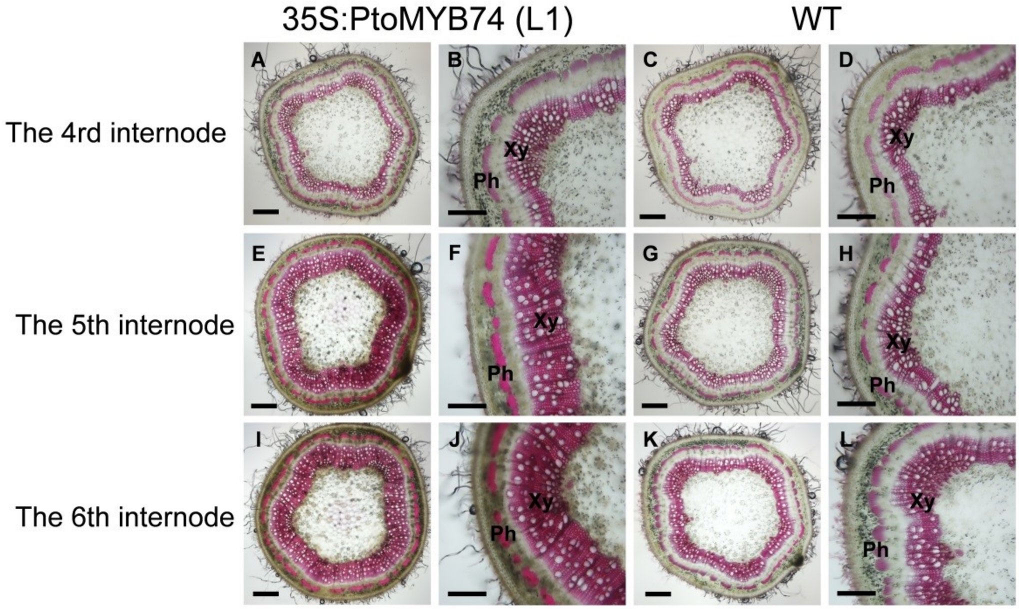

FIGURE S6| Effects of *PtoMYB74* overexpression on secondary cell wall thickening of poplar stems. Stems from the 4th to 6th internodes of 10-month-old plants were stained with phloroglucinol-HCl. Ph, phloem; Xy, xylem. Scale bars: (A, C, E, G, I, K) 500 µm; (B, D, F, H, J, L) 200 µm.

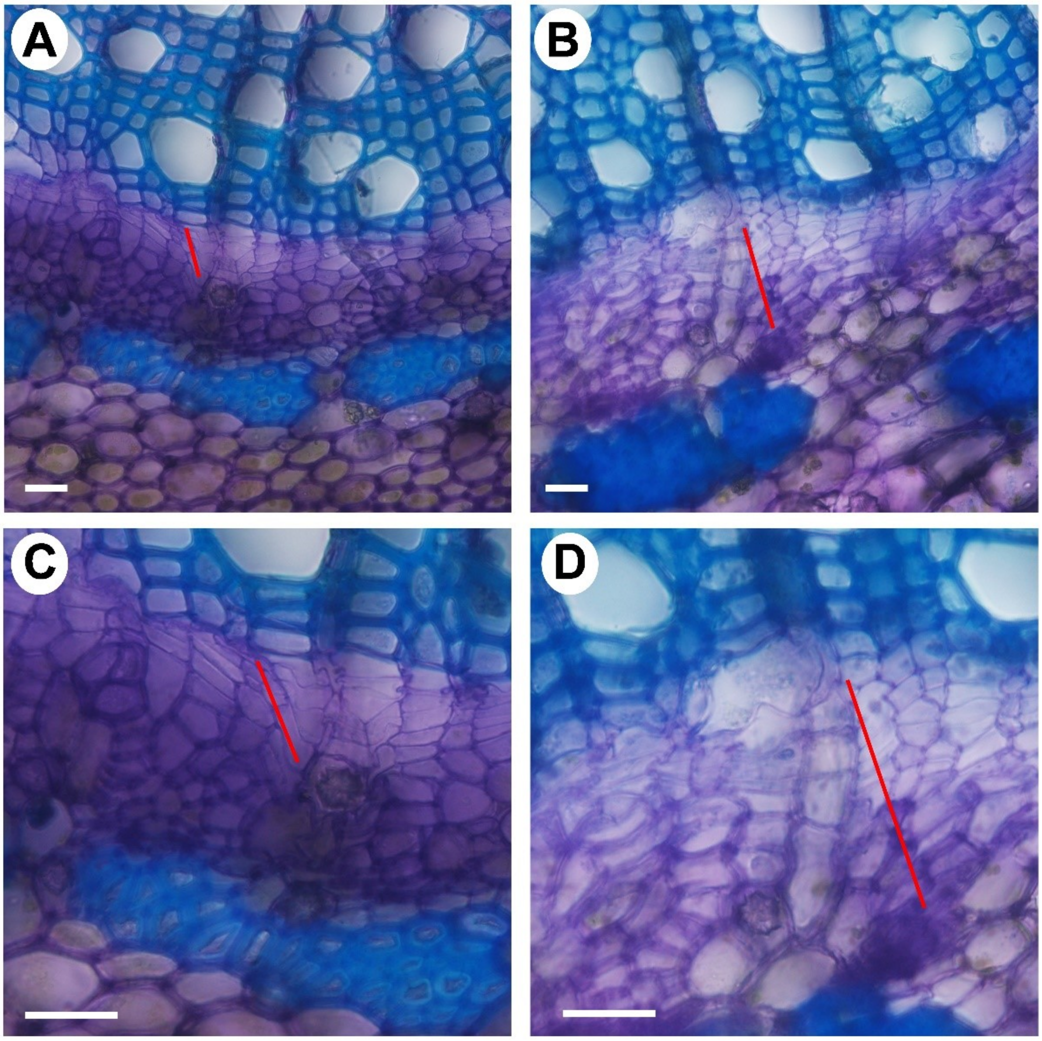

FIGURE S7| Observation of the cambium cells in wild type and *PtoMYB74*-overexpression poplar. Stems from the 6th internodes of 10-month-old plants were stained with TBO. Stem cross-sections used in these images were from the wild type (A, C) and transgenic poplars (B, D). The red lines indicate the location and width of cambium. The cambium cells were indicated with red bars. Scale bars: (A, B) 50 μm; (C, D) 20 μm.

TABLE S1| Primers used in this study.

**Name**	**Forward Primer sequence**	**Reverse primer sequence**
PtoMYB074(cDNA, 1116bp)	5′-ATGGGACGACATTCTTGTTG-3′	5′-TCATATCTGGTGGAAGACAT-3′
HPT （562bp）	5′-CTTCTACACAGCCATCGGTCCAGA-3′	5′-GATGTAGGAGGGCGTGGATATGTC-3′
**Primers for Yeast-One-Hybrid**		
Ye-PtoMYB74 (1126bp)	5′-CCCATATGGGGAGCAGAAATGGGACGAC -3' (NdeI)	5′-CGGGATCCCGGTTCATATCTGGTGGAAGAC-3' (BamHI)
**Primers for Subcellular Localization**		
Sub-PtoMYB74 (1127bp)	5′- AGCAGAAATGGGACGAC -3′	5′-CAGTTCATATCTGGTGGAAGAC -3′
**Primers for GUS activity assay (Promoters)**		
ProPtoMYB074(1562bp)	5′-GCACTAGCTAGGAAGTAGCT -3′	5′-CAACAAGAATGTCGTCCCAT -3′
ProPtr-CCOAOMT1（2005bp）	5′-TGTAGCTTACTTAGTTAGCATACG-3′	5′-GTGTATATCTTCTAATTAAACTAC-3′
ProPtrCCR2（1650bp）	5′-CCGCTCACCACCACAGCTCCTCCAACT-3′	5′-GGGAAAGTGGAGAACAGACAGAGAC-3′
ProPtrC3H3 (1537bp)	5′-CTGGCCTACCTACCCACCACCACC-3′	5′-GGAATGGAGTTAGGAATGGAGTTGGTC-3′
ProPtrC4H2 (1882bp)	5′-GAATCTATGAGGCTGATATTGGTACG-3′	5′-GAGGAGGAGAGACTAGGTGGTTGG-3′
ProPtrGT8D(1953bp)	5′-GCTTGAACAAAACAAACTACTTGGATC-3′	5′-GGATCCGGCTATGTCATTATTTGGCAATCAC-3′
ProPtrGT43B(2024bp)	5′-ATATTCTCAACACTATAGTCATTG-3′	5′-GCTAAACCCCTCAAAAACGTG-3′
Pro-PtrCesA2B (1852bp)	5′-GGAGAGGCTACACTGTCCAGATGGAG-3′	5′-CTGGCTTCCATGTTGAGCAATGG-3′
ProPtrF5H2 (1659bp)	5′-GCTGACTATAGTTCTTGGACGTG-3′	5′-GGATGGTTTATGTGTTGGTG C-3′
**Primers for qRT–PCR**		
PtoMYB07	5′-TGCAGCAGCTTTCTCATCAT-3′	5′-TCATATCTGGTGGAAGACAT-3′

4(259bp)		
Ptr18S (229bp)	5′-GGCATGGAAGGTGATGCAGATC-3′	5′-CTGTGTCAAACAAGAACTTGTCC-3′
AtUBC(101 bp)	5’ GCTGCTATCGATCTGTTCTTTG3’	5’ ACTCGTACTTGTTCTTGTCAGT3’
PtrActin (386bp)	5′-CCGCTCGAGGTTGGACTCTGGTGATGGTG-3′	5′-CCGGAATTCAGTTGTATGTGGTCTCGTGG-3′
Ptr-CCOAOMT1(161bp)	5′-CAAGAGGTTGATTGAGCTTG-3′	5′-GGTCAGCAGCAAGTGCCTTG-3′
PtrCCR2 (276bp)	5′-CTGTTCAAGCTTATGTGCATG-3′	5′-GTGGAGAACGCTCTCAGAGC-3′
PtrCOMT2 (277bp)	5′-CATGAAGTGGATATGCCATG-3′	5′-GTTGAATGCACAGCACATTAC-3′
PtrC3H3 (225bp)	5′-GAGGTTCCTGGAGGAGGATG-3′	5′-GGAGTCGTCATGTAAGTGAC-3′
PtrPAL4 (241bp)	5′-CCTACATTGACGATCCTTGCAG-3′	5′-GACCTGCATTCCTTGATCCTG-3′
PtrHCT1 (110bp)	5′-ATCAGCATGTAAGGCACGCGG-3′	5′-TGCCAAAGTAACCAGGTGGAAGCGT-3′
PtrC4H2 (269bp)	5′-GAGCAAGATCCTGGTAAACGC-3′	5′-CTGAGGTGTCAATCTTGGACTG-3′
PtrCAD1 (233bp)	5′-CAAGCTGATCTTGATGGGTG-3′	5′-CGAATCTATATCTCACATC-3′
Ptr4CL5 (235bp)	5′-CATCCGAGGTGATCAGATCATG-3′	5′-CACAGCAGCATCAGATATCC-3′
PtrF5H2 (221bp)	5′-GAGTCCAGCAAGAGCTCGCAG-3′	5′-GCATAAGCATTGATCATCAC-3′
PtrCesA2B (247bp)	5′-AGGTTAAGATGGAGCGG-3′	5′-ACGAGGTTGATGATCAAGCC-3′
PtrCesA3A (242bp)	5′-CGGATATGGATCATGGGGTCC-3′	5′-GGATAGAGATGGACAATGAC-3′
PtrGT8D (282bp)	5′-GTGCCTGGGCTTATGGCATG-3′	5′-CTAGCCAAGGCTTTGCTCGACC-3
PtrGT43B (264bp)	5′-CTCAATCCTCTGGGATCCTG-3′	5′-GTCTCGTCTTCAAGAGCTAC-3′
PtrGT43D (298bp)	5′-GGTAGAGCCACTTGGGAGC-3′	5′-CACAAGGCAGTATCTCTG-3′
PtrCSE1 (207bp)	5′-GACAGTCCACGGCACGGCTG-3′	5′-CCTCTCAACCCTCTCGTC-3′
PtrWND2B (224bp)	5′-GGCCAGGCTGAAACCTCTAGG-3′	5′-GGCAAGTGCCATTGTGGGTC-3′
PtrWND6B	5′-CCAGCTTGTTGAACTCCCAG-3′	5′-GCTGGCCAGCTGCTAGGTC-3′

(248bp)		
PtrMYB3 (217bp)	5′-GCCTGCAGTCATCCAACGC-3′	5′-CTCGAGTGGCGGAAGAG-3′
PtrMYB20 (361bp)	5′-GAGACCTGACCTCAAGCG-3′	5′-GTTGAGCATGGAGAAAG-3′
PtoMYB92 (149bp)	5′-CGAATACTAACGACGACACG-3′	5′-GTGTTCCATCTCTAATGTGC-3′
PtrMYB128 (189bp)	5' -GATGCAATCCTGTAGCATG-3′	5′-GAGAGTGTAGCAGCTCCCA-3′
PtrMYB148 (209bp)	5' TGACCACGGAAGCATGAGTG 3'	5' GTCCAGCATCATGCCATGGAC 3'
PtrMYB152 (163bp)	5′-GAAGACTTGCTACTGCCAGAT-3′	5′-TCATTCTTGAGCACTGATTG-3′
PtrMYB161 (324bp)	5′-CGAGCAGAAACCTTCCTTCTC-3′	5′-CCAGCCCTGCTCTTAGGC-3′
PtrMYB192 (283bp)	5′-CGTGACCGAAATCCCATTCGAG-3′	5′-GAGTGGCATGTCATGCTG-3′
PtoMYB216(213bp)	5′-CATCTCAACATGTGTACAGTG-3′	5′-GCAGATCCTTGGTATAGATGT-3′
PtrKNAT7 (195bp)	5′-CTACCGGGTGACACTAC-3′	5′-GACCTGTAACTGGTTG-3′
PtrNAC150 (227bp)	5′-CGAAATCCAGCAACAGCAGC-3′	5′-CTACCCATGATGATCCTGG-3′
PtrNAC156 (249bp)	5′-CCACTCTTGTCGAGTAC-3′	5′-CTTCTCAATGTATCCTGCC-3′
PtrNAC157 (178bp)	5′-GAACAGTACAGGCCTCC-3′	5′-CTGCTTTCTCTGAAGC-3′

TABLE S2| GenBank accession numbers used in this study.

Name	GenBank accession numbers	Name	GenBank accession numbers
PtoMYB74	KX887329.1	PtrCCOAOMT1	EU603307.1
PtrCCR2	EU603310.1	PtrCOMT2	EU603317.1
PtrCSE1	XM_002298082.2	PtrC3H3	EU603301.1
PtrPAL4	EU603322.1	PtrHCT1	EU603313.1
PtrC4H2	EU603302.1	PtrCAD1	EU603306.1
PtoMYB216	JQ801749.1	Ptr4CL5	EU603299.1
PtrF5H2	EU603311.1	PtrCesA2B	JX552008.1
PtrCesA3A	JX552264.1	PtrGT8D	EF501824.1
PtrGT43B	JF518935.1	PtrGT43D	JF518937.1
AtUBC9	AT4G27960.1	AtUBC	At5G53300.1
AtMYB46	AT5G12870.1	AtMYB58	AF062893.1
AtMYB61	AF062896.1	AtMYB63	AF062898.1
AtMYB83	AT3G08500.1	HvMYB3	X70881.1
EgMYB2	AJ576023.1	PtMYB4	AY356371.1
PtMYB8	DQ399057.1	PtrMYB003	XM_002299908.2
PtrMYB020	XP_002313303.1	PtrMYB028	XP_002307190.1
PtoMYB92	KP710214.1	PtrMYB128	XM_002304481.1
PtrMYB148	XM_002318028.1	PtrMYB152	XM_006372503.1

PtrMYB161	XM_002309735.1	PtrMYB192	XM_002310643.2
PtrWND2B	XM_002302636	PtrWND6B	XM_002325955
PtrNAC150	XM_006371906.1	PtrNAC156	XM_002309731.2
PtrNAC157	XM_011029466.1	PtrKNAT7	XM_002299533.2
AtMYB85	AT4G22680.1	AtMYB42	AT4G12350.1
AtMYB43	AT5G16600.1	PtMYB1	AY356372.1
AtMYB103	AT1G63910.1	AtMYB32	AT4G34990.1
AtMYB7	AT2G16720.1	ZmMYB42	NM001112539.1
ZmMYB31	NM001112479.1	EgMYB1	AJ576024
AtMYB4	AT4G38620.1	PtrMYB221	XM_002306144.3
PtoMYB156	KT990214.1	PtrMYB002	XM_024609600.1
PtrMYB021	XM_024608290.1	ZmMYB46	NM001254930.1
OsMYB46	JN634084.1	PtoMYB170	KY114929.1
AtMYB52	AT1G17950.1	AtMYB54	AT1G73410.1
AtMYB69	AT4G33450.1	PtrMYB74	XM_002321591.3

TABLE S3| Radial width of xylem and cell wall thickness of vessels in stems of WT and *PtoMYB74* transgenic Arabidopsis plants.

Samples		Radial width of xylem (um)	Vessel cell wall thickness(um)
**WT**		349.45 ±9.36	0.83±0.14
	L1	772.12 ±25.08**	1.35±0.21**
35S:PtoMYB74	L6	563.39 ±15.41*	1.07±0.12*
	L11	647.99 ±19.60**	1.22±0.16*
